# Critical insights on fungal contamination in schools: a comprehensive review of assessment methods

**DOI:** 10.3389/fpubh.2025.1557506

**Published:** 2025-06-23

**Authors:** Renata Cervantes, Pedro Pena, Bruna Riesenberger, Margarida Rodriguez, Drew Henderson, Sara Gonçalves, Enas Newire, Clara Pogner, Heidi Salonen, Marina Almeida Silva, Robert M. W. Ferguson, Ulla Haverinen-Shaughnessy, Carla Viegas

**Affiliations:** ^1^H&TRC—Health and Technology Research Center, ESTeSL—Escola Superior de Tecnologia da Saúde, Instituto Politécnico de Lisboa, Lisbon, Portugal; ^2^NOVA National School of Public Health, Public Health Research Centre, Comprehensive Health Research Center, CHRC, REAL, CCAL, NOVA University Lisbon, Lisbon, Portugal; ^3^School of Life Sciences, University of Essex, Colchester, United Kingdom; ^4^Department of Natural Sciences, Faculty of Science and Technology, Middlesex University, London, United Kingdom; ^5^AIT—Austrian Institute of Technology, Vienna, Austria; ^6^Department of Civil Engineering, School of Engineering, Aalto University, Espoo, Finland; ^7^International Laboratory for Air Quality and Health, Faculty of Science, School of Earth and Atmospheric Sciences, Queensland University of Technology, Brisbane, QLD, Australia; ^8^OSEAN—Outermost Regions Sustainable Ecosystem for Entrepreneurship and Innovation, Funchal, Portugal; ^9^Civil Engineering Research Unit, Faculty of Technology, University of Oulu, Oulu, Finland

**Keywords:** Fungi, exposure assessment, schools, IAQ, target fungal pathogens

## Abstract

This review addresses the increasing problem of fungal contamination in schools, which has a profound impact on indoor air quality and student health. Fungal contamination creates health problems such as respiratory problems, allergies, which can be particularly harmful in schools (e.g., *Aspergillus fumigatus* and *Fusarium* sp. are especially important as they are a well-known indoor allergens and can induce serious respiratory diseases). The aim of this study is to determine the effect of geographic location as well as season of filamentous fungi in school context. Through a comprehensive screening of 6,659 articles, 47 studies were selected for data extraction, detailing sampling techniques, analysis methods, climatic conditions, and relevant fungal species. The study highlights the importance of regularly measuring IAQ and utilizing both active and passive sampling methodologies in addition to molecular genetic analysis to complement identification and improve comparability across studies. A targeted monitoring is also proposed for species such as *Aspergillus fumigatus* (*Aspergillus* section *Fumigati*), *Fusarium* sp., and Mucorales order, which are therapeutically relevant, as well as *Stachybotrys atra* and *Aspergillus* section *Flavi*, in terms of their toxicological potential. Additionally, the article discusses the importance of consistent data formatting for effective meta-analysis and the need for further research to inform regulatory frameworks protecting student health. Recommendations for minimizing fungal threats include evaluating building structure, ventilation, cleaning practices, and gathering information from parents about school activities. Overall, the study underscores the global health risks posed by fungi in schools and calls for extensive investigations combining various sampling and analytical techniques. Additionally, the article discusses the importance of consistent data formatting for effective meta-analysis and the need for further research to inform regulatory frameworks protecting student health. Recommendations for minimizing fungal threats include evaluating building structure, ventilation, cleaning practices, and gathering information from parents about school activities. Overall, the study underscores the global health risks posed by fungi in schools and calls for extensive investigations combining various sampling and analytical techniques.

## 1 Introduction

In 2022, the Health Emergency Preparedness and Response Authority (HERA) presented a priority list of top-3 health threats that require coordination of measures at the EU level, since they have the potential of spreading across Member States. All three health threats highlight the importance of microbes and stress the need to assess exposure to: pathogens with high pandemic potential; chemical, biological, radiological and nuclear threats; and threats resulting from antimicrobial resistance.^1^ Also in 2022 focusing on fungi, the World Health Organization (WHO) release a list of fungal priority pathogens focusing on their clinical relevance to guide research, development, and public health action (1). The list is divided into three groups: critical, high, and medium priority groups and the ones listed are mostly important due to their clinical relevance (1). However, the concern regarding the toxigenic potential of specific fungal species and strains was not considered, hindering a more accurate intervention when assessing IAQ.

A warmer, wetter climate driven by human induced climate change is driving range shifts, increased dispersal, and the emergence of new fungal pathogens (2). In addition, many of the antifungals (e.g., azoles) used in clinical settings are also used in crop protection fostering azole resistance among fungal species (3, 4) also found in various indoor environments (5, 6).

A variety of regulations exist for microbial and chemical pollutants in indoor spaces, with the goal of enhancing indoor air quality and health. An open database was created by a scientific committee (7) to gather and distribute information on indoor environmental quality (IEQ), containing guidelines and standards from numerous countries and organizations (8). Overall, the guidelines and standards assembled in the IEQ guidelines (9) database present a great diversity, complexity, and inconsistency not only among countries but also within countries. Table 1 summarizes the database regarding fungal colony-forming units (CFU).

**Table 1:** Summary table regarding international fungal exposure guideline values (7), focused on indoor air quality (IAQ) parameters (8).

Country (nation with regulations)	Parameter (mold or moisture issue)	Threshold (acceptable contamination level)	Criteria (evaluation method or risk classification)
South Korea	Mold	500 CFU.m^−3^	Mixture of species
United Arab Emirates	Mold	500 CFU.m^−3^	Mixture of species
Singapore	Mold	<500 CFU.m^−3^	>500 CFU/m^3^ if predominant species is *Cladosporium* spp.
Malaysia	Mold	1,000 CFU.m^−3^	Mixture of species
Brazil	Mold	750 CFU.m^−3^	Mixture of species
	Mold	Indoor/Outdoor ratio < 1.5	Mixture of species
Spain	Mold	200 CFU.m^−3^	Mixture of species
Belgium	Mold	<50 CFU.m^−3^—very low risk <200 CFU.m^−3^—low risk <1,000 CFU.m^−3^—medium risk <10,000 CFU.m^−3^—high risk >10,000 CFU.m^−3^—very high risk	Mixture of species
Portugal	Mold	Indoor/Outdoor ratio < 1	Mixture of species
Norway	Dampness	Visible mold damage or odor of mold	
United Kingdom	Dampness	Visible mold on external walls in a properly heated dwelling	
Finland	Dampness	Unrepaired moisture/rot damage on the inner surface, internal structure, or thermal insulation of a building	
Denmark	Dampness	0 cm^2^—habitation/occupable room 400 cm^2^—wet rooms 2,500 cm^2^—roof spaces and basements	

Specifically for fungi in indoor air, the database includes guidelines from 12 countries. Eight countries have numerical limit values but there is little consensus, with values ranging from as little as 50 CFU/m^3^ to 10,000 CFU/m^3^. The large range of values reflects the current lack of an established dose–response relationship between concentrations of airborne fungi and health outcomes. Due to the lack of a scientific basis for defining numerical health-based values for indoor fungal concentrations, many guidelines are based on the assessment of dampness and mold, as these have been most consistently associated with adverse health outcomes, including respiratory conditions such as asthma, rhinitis, and other respiratory tract infections, particularly in vulnerable populations like children, individuals with pre-existing respiratory conditions, and those with immune deficiencies (10). Indeed, WHO (11) has concluded sufficient epidemiological evidence from studies conducted in different countries and under different climatic conditions to show that the occupants of damp or moldy buildings, both residential and public buildings, are at increased risk of respiratory symptoms, respiratory infections, and exacerbation of asthma. The definition of dampness and mold based on the WHO guidelines is “any visible, measurable or perceived outcome of excess moisture that causes problems in buildings, such as mold, leaks or material degradation, mold odor or directly measured excess moisture (in terms of relative humidity or moisture content) or microbial growth.” The WHO has also proposed data collection regarding dampness in buildings via inspections in schools in the WHO region (12). Reflecting this, many countries set guidelines based on visible inspection only (e.g., Ministry of Social Affairs and Health, Finland, 2015), (Danish Enterprise and Construction Authority, Copenhagen 12. Of December 2010).

While establishing upper limits based on clear links between exposure and health outcomes is critical, it is unlikely that it is optimal to eliminate exposure to fungi in indoor air entirely. As postulated in the “Hygiene” hypothesis, as humans co-evolved in the presence of various microorganisms, they may play a significant role in the regulation and childhood development of the immune system (13). This would suggest that increasing rates of inflammatory disease (such as asthma) with urbanization may be partly explained by reduced exposure to microorganism diversity and parasites in childhood as humans moved away from rural lifestyles in the mid-19th century. In the context of airborne molds/fungi, this could be used to support the argument that thresholds for airborne fungi in schools should not be zero and that some sort of “Goldilocks Zone” of exposure should be established. However, it is known that exposure to some viral and bacterial respiratory infections does not provide protection against asthma, indeed the opposite has been observed (14, 15).

Currently, established dose–response relationships between concentrations of airborne fungi and health outcomes are limited and complex. Research has shown clear associations, particularly in allergic and respiratory conditions such as asthma and allergic rhinitis, where exposure to high levels of fungi like *Aspergillus*, *Cladosporium*, and *Penicillium* exacerbates symptoms (16) however, specific dose–response data are often unclear due to variations in individual sensitivities and environmental factors. Immunocompromised individuals are known to be at greater risk for fungal infections at higher exposure levels, yet precise thresholds remain undefined (17). Fungal contamination in schools has potential negative outcomes on both the health and learning ability of students (18). The presence of fungi such as *Aspergillus*, *Penicillium*, and *Cladosporium* in school environments can lead to a range of health issues, including respiratory problems, allergies, and asthma exacerbation. Moreover, fungal contamination may compromise the structural integrity of buildings and contribute to indoor air quality degradation (11). Beyond health implications, the presence of fungi in schools can significantly impact students’ concentration, cognitive function, and academic performance (19, 20). Furthermore, prolonged exposure to fungal toxins may result in chronic health conditions, exacerbating absenteeism rates among students and educators (21, 22). Fungal contamination in schools poses significant health risks, including respiratory issues, allergic reactions, and potential infections. Since fungi can enter the body through airways, food, and water, it is crucial to emphasize the importance of regular monitoring and mitigation strategies (23). Currently, addressing fungal contamination lacks comprehensive strategies, including regular inspections, effective moisture control, and prompt remediation, all essential to safeguard health and optimize educational outcomes. It also needs to be noted that decreased exposure to beneficial microorganisms is not the only driver of inflammatory diseases, for example, increased exposure to particulate air pollution is a key contributor. The focus needs to be specifically on the now absent beneficial microorganisms. The specific fungal microbiome that is helpful for the human immune system cannot be replaced with new microorganisms that we did not co-evolve with. It is therefore critical to define the principal airborne fungi, we should (and in particular children with developing immune systems) be exposed to along with the numerical concentrations.

Despite this, there is still a lack of *consensus* on how to assess exposure to fungal contamination indoors, hampering the possibility of comparing results and identifying suitable fungal “sentinels” specific to each indoor environment. As such, it is of utmost importance to identify the best protocol regarding sampling collection and analyses regarding fungal exposure assessment, as well as to identify the most suitable fungal targets for the school environment (24). Although the literature reports a wide range of sampling methods and assays currently applied to assess fungal contamination indoors, there is no harmonized evaluation or even common approach regarding IAQ assessment among researchers, even when complying with the legal requirements. Thus, the aim of this scope review was to identify the methods used for fungal sampling and analyses and to list the fungal species that can be suggested as targets to assess IAQ regarding fungal contamination. The results retrieved from studies may contribute to setting future protocols (from the field to the lab) to assess fungal contamination in schools and to identify indicators of harmful fungal contamination for this specific environment. Furthermore, we are stressing the importance of a *consensus* regarding exposure assessment and considering the potential health effects due to exposure. This work will be also important to ensure both an accurate risk characterization and, consequently, the suggestion of effective control measures.

## 2 Materials and methods

Give a comprehensive overview of the literature available on the topic described above, a systematic literature review of studies was performed to identify the sampling strategy, methods used for fungal sampling and analyses applied and to list the fungal species that can be suggested as targets to assess IAQ regarding fungal contamination.

To aid in the identification of search terms and inclusion/exclusion criteria required to address this systematic review, a PEO (Population, Exposure and Outcome) (104, 106), statement was developed by the authors´ team (Supplementary Table S1).

### 2.1 Registration

In this study, the PRISMA methodology was adopted and the Preferred Reporting Items for Systematic Reviews and Meta-analysis (PRISMA) checklist (107) was completed, which encompass three phases: Identification, Screening, and Included (25).

### 2.2 Search strategy, inclusion and exclusion criteria

This study reports the search of available data published between January 1st, 2010, and February 29th, 2024. The search aimed at selecting studies on fungal assessment in different indoor school environments and included the terms presented in Supplementary Table S2, with English as the chosen language. The databases chosen were PubMed, Scopus, and Web of Science (WoS). Articles that did not meet the inclusion criteria and duplicates were excluded from further analysis (Table 2).

**Table 2:** Inclusion and exclusion criteria in the articles selected.

Inclusion criteria	Exclusion criteria
Articles published in the English language	Articles published in other languages
Articles published from 1st January, 2010	Articles published after February 2024
Articles published in any country	
Articles related to IAQ in elementary schools.	Articles related to IAQ in elementary schools, without mention the fungal contamination.
Articles applying all types of sampling methods	Articles related to other school years (not elementary)
Original scientific articles on the topic	Abstracts of congress, reports, reviews/state of the art articles

### 2.3 Studies selection and data extraction

The selection of the articles was performed through Rayyan, which is a free web tool that greatly speeds up the process of screening and selecting papers for academics working on systematic reviews. The screening was performed in three rounds by two investigators (RC and PP,). The first round consisted of a screening of all titles to exclude papers that were duplicated or unrelated to the subject and subsequent adding of included papers to Rayyan for further analysis. The second round consisted of a screening of all abstracts and in the third round, the full texts of all potentially relevant studies were reviewed considering the inclusion and exclusion criteria. Potential divergences in the selection of the study were discussed and ultimately resolved by the remaining investigators who contributed to this study. Data extraction was performed by five investigators (RC, PP, DH, EN and SG) and reviewed by the other two (CV and MS). The following information was manually extracted: (1) Database, (2) Title, (3) Country, (4) Occupational Environment, (5) Climate region, (6) Sampling sites, (7) Environmental samples description, (8) Sampling methods, (9) Analytical methods, (10) Fungi targeted, (11) Observed concentrations (CFU/m3 or other units depending of the applied methods), (12) Components/metabolites, and (13) reference (Supplementary Table S3). The prevalence analysis was based on the number of studies that identified each fungal genus or species. A higher proportion of studies reporting a particular genus or species indicates its more frequent detection in the selected literature.

## 3 Results and discussion

**Figure 1:**
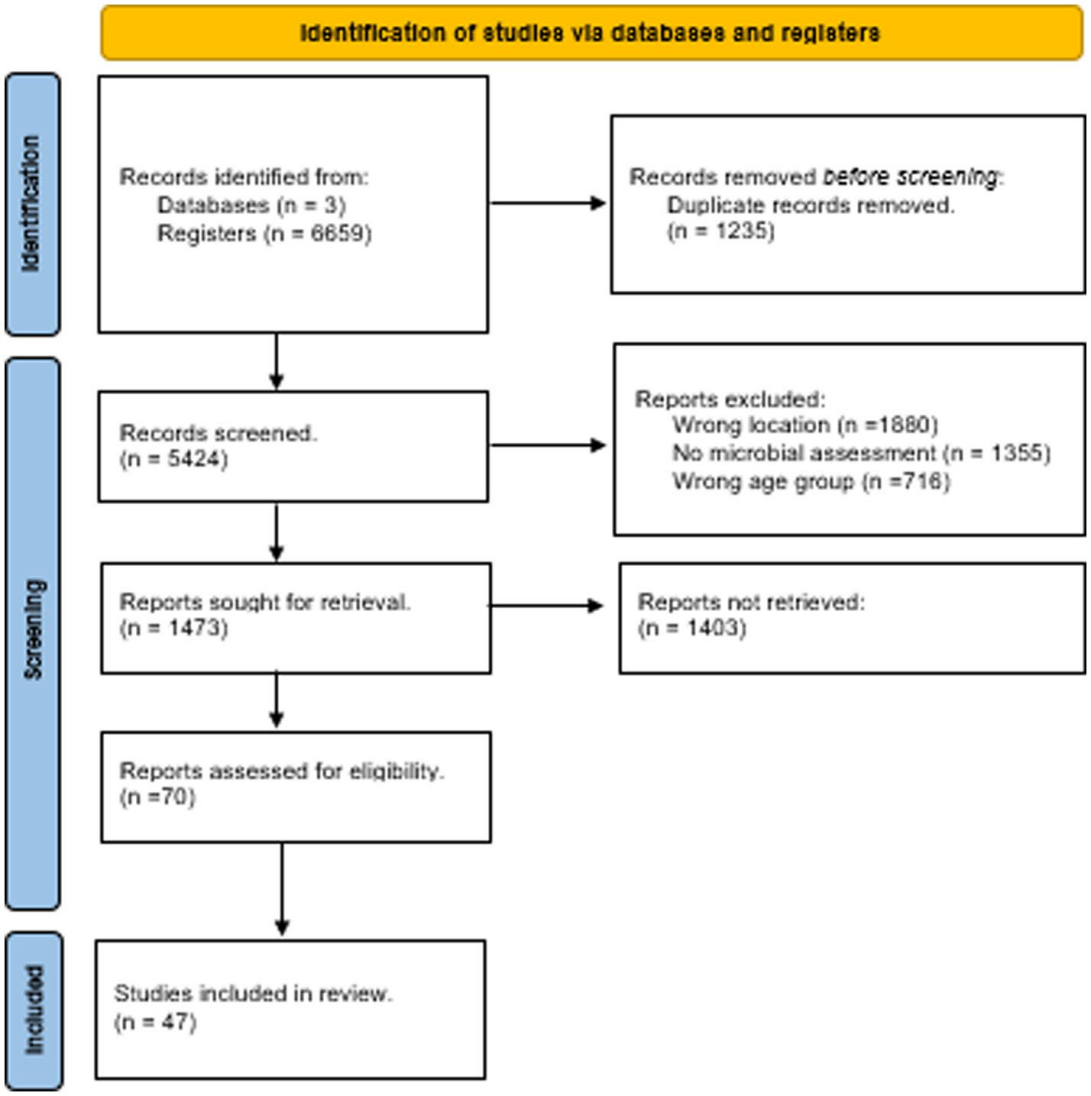
PRISMA based selection of articles (73).

In the systematic search for papers, multiple combinations of search terms were used in every round of the search. Each time the terms “school,” “children,” and/or “indoor air” were used and, at least three different terms were combined at once. Literature reviews were excluded from the search. The diagram describes the different phases of the selection of papers and the papers that were obtained in the final phase (Figure 1). The initial database search yielded 6,659 studies, from which 1,473 titles and abstracts were examined, and 101 full texts were evaluated for eligibility. A total of 3,902 studies were rejected after examining the inclusion and exclusion criteria, primarily because they were related to chemical evaluation, not performed in schools, or others (viruses, bacteria, reviews, surveys, etc.). After evaluation, a total of 70 papers on fungi in school environments were selected, from which 47 papers were eligible for data retrieval. The excluded papers did not meet the inclusion criteria, as they lacked essential details such as species identification, methodology, or other critical information required for the analysis.

In this comprehensive review, we encompassed rigorous evaluations of sampling methods, analytical techniques, and climatic conditions across different seasons. The outcomes disclosed in this document are organized in a graphic configuration, focusing on essential aspects such as the country of study, specific sampling sites, employed sampling and analytical methodologies, the identified agents, and the prevailing climate or seasonal conditions. The reported health effects, other associated problems, and study limitations have been meticulously documented, providing a holistic overview of the intricate relationships between environmental variables and human health outcomes. Understanding indoor microbial communities requires a broader perspective that includes interactions between fungi, bacteria, and viruses. Studies employing vacuum dust collection and sequencing methods have provided valuable insights into the total microbial composition in school environments, linking these multi-kingdom microbiomes to respiratory health outcomes. While such studies offer a more integrated approach to microbial exposure assessment, they fall outside the scope of this review due to differences in selection criteria, which focus specifically on fungal contaminants. However, acknowledging the complex interplay between different microbial groups is essential, as their combined presence may have synergistic or antagonistic effects on indoor air quality and health. Future research integrating diverse microbial communities could provide a more comprehensive understanding of exposure risks in school settings.

### 3.1 Spatial distribution of sampling sites

**Figure 2:**
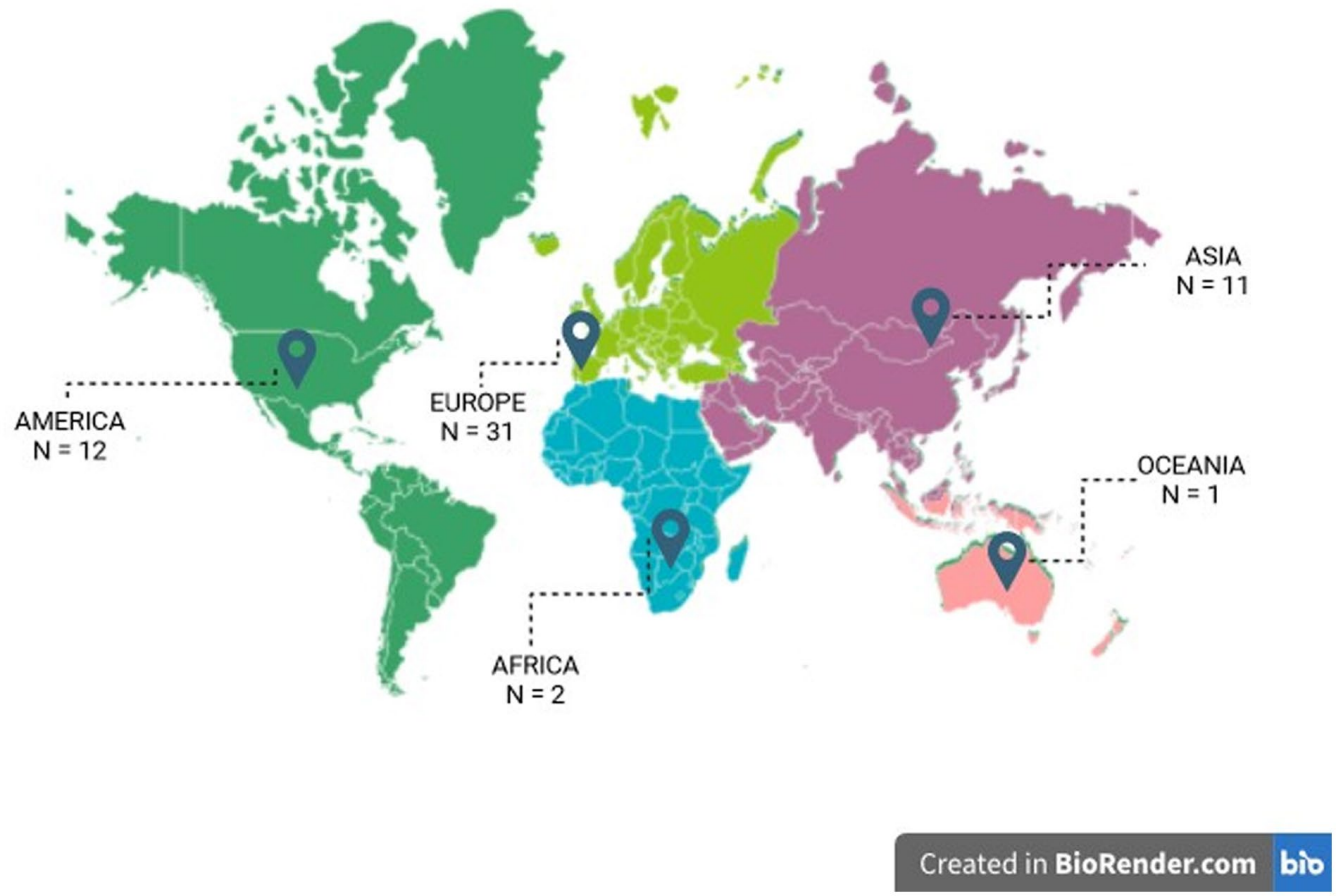
World map showing the geographic distribution of the studies.

The distribution of research efforts across various geographical regions is crucial for understanding school indoor air quality (IAQ). Among the 47 studies assessed, data from 25 countries were included. Some of the studies were performed in more than one country (Figure 2). Notably, a substantial proportion of studies, accounting for 54% of the overall dataset, have been conducted on the European continent, followed by America (21%) and Asia (19%) (Figure 2). This proportion may be attributed to factors such as ecological significance, accessibility, or the presence of unique environmental features relevant to the research domain (26), or also due to the resources available and the focus on air quality and health. But it also represents a significant blind spot in our understanding, for example only 2 of the studies were conducted in Africa, and 3 in the Global South.

### 3.2 Seasonal effects

The examination of the climatic aspect within the analyzed studies indicates a significant focus on considering climate conditions during the sampling process. Out of the total 47 studies, 33 refer to seasonal variations, with 17 of them implementing a bi-seasonal approach, covering two distinct periods of the year and 16 mentioning the selection of a specific time frame (either warm or cold season). The selection of one season shows a predominance of data collection during the cold seasons, indicating a purposeful decision influenced by the particular environmental characteristics of these periods (13 out of 47). The preference for these specific periods may have implications for the interpretation of study results, as environmental factors can significantly influence outcomes. To enhance the temporal understanding of the subject matter, five longitudinal studies were included reporting durations of 1 and 2 years which provide a comprehensive perspective on how variables evolve, offering valuable insights into trends and patterns, and the dynamic interplay between geographic and climatic factors over extended periods, that may not be evident in shorter-term studies (27).

Airborne fungal concentrations vary based on factors like season, location, outdoor levels and ventilation. Studies show that indoor fungal concentrations can range from low to high levels, with values typically above 500–1,000 CFU/m3 being a cause for concern, indicating possible building contamination (28). Specific genera like *Cladosporium*, *Penicillium*, *Aspergillus*, and *Alternaria* have been linked to health effects like asthma exacerbations, with concentrations fluctuating based on temperature, relative humidity, and particulate matter levels (29, 30). Additionally, research highlights the importance of assessing fungal exposure in different environments like homes and schools, with concentrations being notably higher in winter compared to summer in certain regions (31, 32). Significant variations between cold and warm seasons in indoor environments have been observed in multiple studies. Additionally, fungal spore richness differs between seasons, with Basidiomycota richness higher in hot seasons and Ascomycota richness higher in cool seasons (33). During the warm season, insights contribute to designing strategies for managing environmental quality in schools (34). In contrast, the winter season shows higher indoor fungal growth due to closed windows and poor ventilation, leading to increased bioaerosol concentrations with rising indoor temperatures (35). Additionally, indoor *Cladosporium* levels are affected by outdoor conditions, with higher concentrations in winter due to low humidity and high wind speeds (32).

### 3.3 Sampling location

In the vast majority of the studies, the process of sampling was conducted within the confines of the classrooms (38 out of 47). However, it is worth noting that numerous studies also conducted sample collection in various areas of the school building, such as the canteens, corridors, and even on surfaces, such as walls, working tables, and floors and some outdoor samples were also collected in some of the studies.

The findings presented show the profound variation in used sampling approaches and the need for the inclusion of contextual information in research studies. Given that fungi exposure is influenced by multiple factors, it is crucial to describe all relevant information in the publications and consider the context of the study design in the interpretation of the results to achieve accurate exposure assessment. The collection of information regarding building structure, ventilation, cleaning practices, and the activities description allows the identification of hazards (36, 37). The application of a walkthrough survey to collect such information allows a comprehensive understanding of the contamination context and supports the implementation of control measures (38). Moreover, the mention of contextual information, including surveys on school operational and structural conditions as well as reports from parents regarding children’s health-related issues, highlights a step toward holistic understanding within research (39, 40). Such data provide valuable insights into the broader circumstances surrounding the research subject, offering a more nuanced interpretation of the findings (39, 40). However, the relatively low percentage (7%) of the reviewed articles including contextual information suggests a need for greater emphasis on contextual factors in research methodologies. In the articles reviewed, the association between mildew presence and increased mold concentrations is emphasized (32, 41, 42), alongside the repercussions observed in moisture-damaged schools, including elevated microbial markers, heightened respiratory symptoms, and higher fungal DNA concentrations (43). Carpeting was associated with the presence of bioaerosols, indicating the importance of regular cleaning and monitoring for signs of dampness or moisture damage (44–46).

### 3.4 Sampling methods

In terms of sampling, the majority (32 out of 47) employ active sampling methods, which involve drawing air through a collection device using a pump, utilizing air samplers, impactors, impingers, and other aerosol devices. In contrast, 15 out of 47 exclusively use passive methods, which rely on natural air deposition onto a surface or medium without mechanical assistance, employing techniques such as settled dust, electrostatic dust cloths, and the open-dish method (24) (Table 3).

**Table 3:** Summary of active and passive sampling methods and respective samplers used in various studies.

Active/passive	Method category	Sampling method/samplers	References
Active	Impaction	Burkard indoor recording air sampler	(30–32, 74, 75)
		Single-stage microbiologic air impactor (Merck MAS-100)	(76, 77)
		Single-stage AirIdeal 3P impactor	(34, 35)
		Andersen N6 single-stage impactor	(28, 78–80)
		Six-stage Andersen 10–800 impactor	(42, 81, 82)
		Mattson–Garvin slit-to-agar impactor	(44)
		Andersen two-stage cascade impactor	(83, 84)
	Filter	IOM inhalable dust sampler (SKC Inc.)	(85, 86)
		Air-O-cell cassette (SKC/Zefon)	(16, 45)
		Nucleopore filters (0.4 μm)	(87, 88)
		Millipore cassettes (0.45 μm)	(89)
		Fine particle sampler (PM2.5 filters)	(44)
		MCE filter cassettes (0.8 μm)	(28, 90)
	Vacuum	Micro-vacuum sampler (IAQ-1294)	(28, 86)
		Siemens Super XS vacuum cleaner	(88, 91)
		Li’l Hummer backpack vacuum	(92)
		HVS-3 vacuum sampler	(86)
		Generic vacuum cleaners	(93, 94)
	Tracer gas	Tracer-gas decay method (acetone)	(87)
	Other	Sampling pumps (unspecified)	(95)
		Spot sampler (aerosol devices)	(90)
Passive	Electrostatic dust collectors (EDC)	Electrostatic dust collectors (EDCs)	(41, 55, 85, 96, 97)
	Settled dust	Settled dust boxes (SDBs)	(43, 85, 86, 96, 105)
		Passive sedimentation (open-dish/gravity plates)	(23, 98–101)
	Swab/surface sampling	Surface swabs	(23, 101–103),

With only 7 out of the 47 studies utilizing both active and passive methods for sampling, there’s a clear opportunity for improvement in achieving a more comprehensive understanding of climate change and seasonal sampling impact. Active sampling requires a pump, collecting a specific volume of air for a defined period by the researcher/exposure assessor. Air can be collected onto culture media, liquid media, or any kind of membrane or filter (polyvinylchloride, polycarbonate, cellulose acetate, or gelatine filters). These methods allow the application of culture-based methods (allowing the identification of viable particles) (47–49). On the other hand, passive sampling methods do not require a pump or any other mechanical equipment. They are based on settled dust collection onto a Swiffer, agar plate, filter, or swabs. They allow sampling for longer periods, as they enable the accumulation of dust over extended durations, such as days, weeks, or even months, providing a more integrated representation of airborne fungal exposure. However, the sample needs to be extracted through a liquid solution for subsequent use of a culture-based method (allowing the identification of viable particles) and molecular tools, as in the case of Electrostatic Dust Cloths (EDC) (3, 49). By using both sampling approaches, researchers can capture a wider range of perspectives and nuances, potentially enhancing the validity and reliability of their findings (50). The articles present their results using disparate methodologies, with many offering only statistical data that are difficult to compare with those from other studies, thereby limiting the scope for broader analyses.

### 3.5 Analytical methods

Among the studies that were examined, a substantial proportion (32 out of 47) chose to use culture-based methods with macroscopic and microscopic observations as part of their analytical approach. This conventional methodology enables researchers to directly visualize the morphological characteristics of both colonies and fungi, thus facilitating thorough examinations (51). However, it is well documented that only a small percentage of fungi recovered from air samples are culturable. Thus, using culture-based methods may lead to an underestimation of the real fungal contamination (52, 53).

However, a significant subset of the studies (11 out of 47) exclusively employed molecular genetic assays for analyzing their samples. Molecular genetic techniques, such as qPCR and DNA sequencing, offer robust mechanisms for detecting and characterizing genetic material with high sensitivity and specificity (54). Nevertheless, these methods have limitations, including detection bias, inability to assess organism viability or morphology, reliance on amplifiable DNA, and high costs (52, 54). Notably, one study (55) combined culture-based methods with molecular techniques (qPCR and antifungal resistance testing), leveraging the strengths of both approaches to identify viable organisms and characterize genetic profiles. Meanwhile, 11 studies integrated culture-based methods with biochemical/enzyme assays, providing insights into functional properties like toxicity or allergenicity. The majority of studies (30/47) relied on single-method approaches—either culture or molecular—highlighting a gap in multi-method frameworks that could address the limitations of standalone techniques. These findings underscore the need for future research to adopt hybrid methodologies that balance viability assessment, morphological identification, and genetic specificity for comprehensive risk evaluations. The array of analytical methods observed underscores the significance of having methodological adaptability in scientific investigations. To provide a more nuanced discussion on the strengths and weaknesses of these methods, a larger number of studies utilizing both approaches would be required. Currently, the limited number of studies makes it difficult to definitively address whether there are specific scenarios in schools where one method is clearly preferable or more informative than the other. This progress fosters innovation in research despite some constraints. By combining culture-based observations with molecular genetic assays, researchers can gain a more comprehensive understanding of specimens, uncovering insights that might be missed using only one method. However, comparing culture-based techniques and molecular genetic assays poses challenges. The main issue is aligning macroscopic colony morphology and microscopic identification with genetic data. To overcome this, researchers may need to develop standardized criteria or conduct simultaneous use of culture-based and molecular identification methods on the same sample together to take advantage of their strengths and minimize their individual limitations. Furthermore, since culture-based methods allow the comparison with quantitative cut-offs referred to in many international guidelines (Table 1), integrating molecular techniques for the identification and confirmation of pathogenic species can enhance the accuracy, sensitivity, comprehensiveness, and comparability of assessments, thereby supporting improved risk management and more effective public health interventions (56–59). Furthermore, the screening of azole resistance is dependent on obtaining an isolate from the species under study and thus relying on culture-based methods, at least as first step of the screening (60). Additionally, numerous articles considered in this review included supplementary analyses, including endotoxin and cytotoxicity assessment, as well as antifungal (azole) resistance testing as indicated in Supplementary Table S3.

### 3.6 Most prevalent fungal taxa

**Figure 3:**
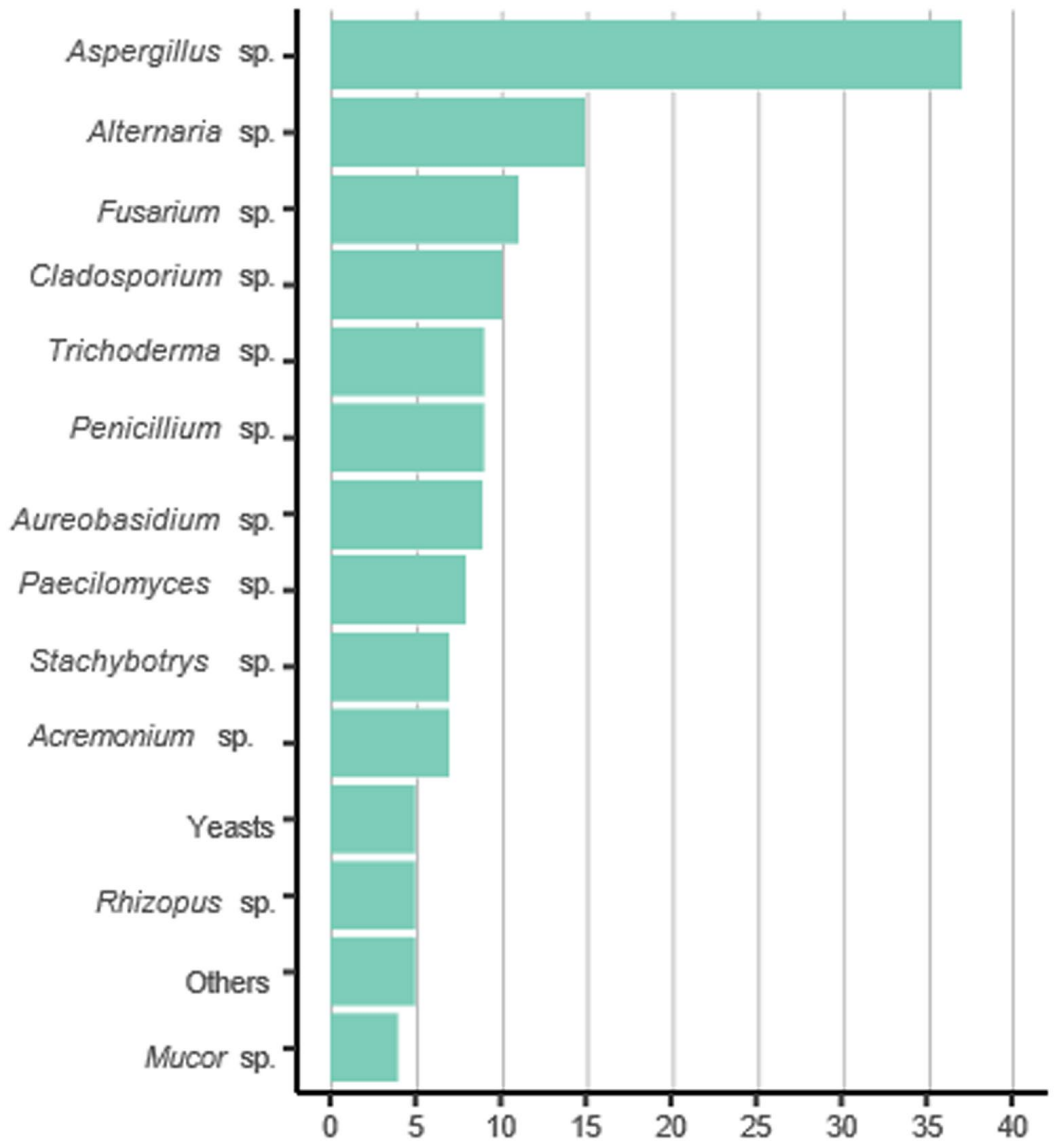
Topmost prevalent genera found in the selected articles.

**Figure 4:**
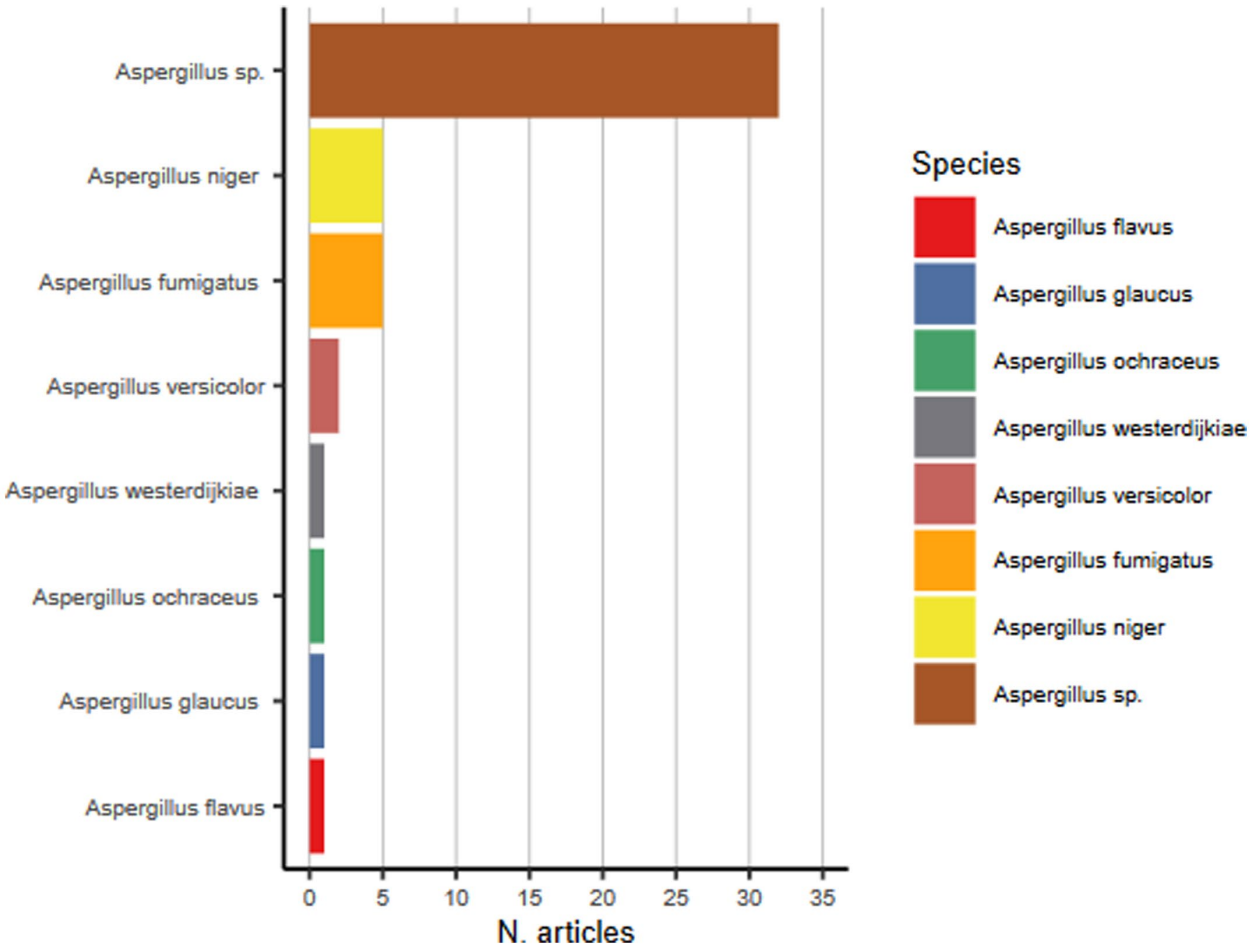
*Aspergillus* species identified in the selected articles.

Figure 3 presents the most prevalent fungal genera identified across the selected studies. The prevalence reflects the number of studies that reported each genus, with higher representation indicating more frequent detection in the literature., among 47 studies analyzed, 42 mention the presence of *Aspergillus* species, of which merely 16 were identified up to the section (Figure 4), and only 5 identified as *Aspergillus fumigatus.* Since *Aspergillus fumigatus* is on the list of priority pathogenic fungi as a critical priority, which is based on criteria such as antifungal resistance, mortality, evidence-based treatment, access to diagnostics, annual incidence and complications and sequelae, it is of the utmost importance to be identified (1). Furthermore, two other high priority species were identified in 20 of the 47 studies, where 11 mentioned the presence of *Fusarium* sp., and 9 identified the presence of Mucorales.

The most prevalent genera was *Aspergillus* (Figure 3). It is noteworthy that among the top 46 identified genera examined in various studies, 11 were predominantly found in indoor settings within elementary schools, (Figure 3). These particular genera encompassed *Aspergillus*, *Alternaria*, *Cladosporium*, *Aureobasidium*, *Penicillium*, *Trichoderma*, *Fusarium*, *Paecilomyces*, *Acremonium*, *Stachybotrys*, *Rhizopus, Mucor* and yeasts. Considering that some genera are easily identified macroscopically, there may be a bias in statistics toward those that have been analyzed. If most studies focus primarily on these genera, this could explain the absence of others in the findings.

Figure 4 summarizes the distribution of Aspergillus species identified in the selected studies. The prevalence is based on the number of studies reporting each species, highlighting the most commonly detected ones either based on optical analysis and/or molecular genetic tools. The most frequently encountered were *Aspergillus niger*, *Aspergillus fumigatus*, and *Aspergillus versicolor* (Figure 4). *Aspergillus* species identification is crucial due to the emerging resistance profile and consequently clinical implications (61). Nowadays, the taxonomic classification of fungi is undergoing a transformation. *Aspergillus* species are classified into taxonomic groups based on their morphological characteristics (62). *Aspergillus* sections may encompass multiple species, and accurate identification often requires a combination of morphological, biochemical, and molecular genetic methods (63). However, more recently, genetic approaches, especially focusing on rRNA genes, have found that many fungi do not fit neatly into the categories used based morphological and biochemical characteristics (64). For example, among the *Aspergillus* genus, the designations represent sections of closely related species (also referred to as cryptic species) that cannot be clearly distinguished morphologically (65). These sections include *Aspergilli, Fumigati, Circumdati, Terrei, Nidulantes, Ornati, Warcupi, Candidi, Restricti, Usti*, and *Flavi*, among others. *Aspergillus* identification to, at least, section level provides a comprehensive understanding of the diversity, ecology, and significance of this genus (66). In fact, accurate identification of *Aspergillus* species within clinically relevant sections informs diagnostic strategies, treatment decisions, infection control measures, and public health interventions (67, 68).

Regarding yeasts, many papers suggest the possibility of yeasts in schools being mainly derived from different parts of the human body, potentially explaining their high concentrations indoors (Supplementary Table S3).

The growth of mycotoxin-producing fungi (e.g. S*tachybotrus atra*) was reported in one study (10). In one study, *Aspergillus* concentrations indoors correlated significantly with the concentration of particulate matter (16), and in one study dust was reported as a major contribution to fungal and bacterial flora in schools (18). Also the age of buildings, area of classrooms, temperature, humidity, and particulate matter (PM10) were reported as predictors of the concentration of culturable fungi (19). Finally, it was suggested that increased ventilation rates may mitigate overheating, alleviate sick building syndrome symptoms, and improve satisfaction with IAQ (28). All the studies underscored the importance of routine testing for fungal contamination in schools and emphasized the need to address poor ventilation to mitigate health risks such as allergies and respiratory illnesses.

The comprehensive summary of the found species in school environments shows that it is crucial to highlight the prioritization of research and evaluation efforts regarding indoor air quality in school health programs, with an emphasis on investigating, quantifying, and recognizing fungal pathogens with significant clinical implications on human health, as the ones listed in the WHO priority list and in Table 4. Since the toxicological potential of the fungal species was not considered in the WHO list, we also recommend the inclusion of mycotoxin-producing fungi, such as *Stachybotrus atra* and *Aspergillus* section *Flavi.* The latter is the main producer of the carcinogenic mycotoxin Aflatoxin B1 (AFB1), which is predicted to increase due to climate changes in Europe (69–71), as rising temperatures, humidity, and extreme weather events create favorable conditions for fungal proliferation—trends that are also observed globally in regions experiencing similar climatic shifts (72).

**Table 4:** Comparative analysis of WHO fungal priority pathogens list and other pathogens with health impact.

WHO fungal priority pathogens list (WHO FPPL)		Pathogens identified in the reviewed papers (*n*)	Paper references
Critical Priority group	*Aspergillus fumigatus*	5	(28, 35, 76, 81, 83)
High priority group	*Fusarium* spp.	11	(23, 28, 30, 32, 35, 75, 83, 97–99, 101)
	Mucorales	9	(23, 28, 34, 76, 83, 91, 97, 99, 102)

Papers reviewed are listed in the last column, providing a reference for further studies and analysis.

## 4 Conclusion

In summary, understanding and monitoring fungal concentrations is crucial for assessing indoor air quality and potential health risks, as geographic distribution, climatic conditions, and outdoor air significantly influence fungal diversity and activity. Given that much of the research has focused on specific regions and climate periods—resulting in a biased understanding of the implications for human health—and that most studies rely on active sampling and culture-based methods, which form the cornerstone of fungal contamination assessment, their complementary integration with passive sampling and molecular genetic tools can significantly enhance the overall approach. Additionally, the comparability of fungal contamination data depends on detailed contextual information, including anthropogenic activity, structural information, ventilation, and cleaning practices. Overall, we recommend:

- Standardized Protocols: Develop standardized protocols for sampling and analysis to ensure consistency in fungal assessments across studies.
- Integration of Molecular Methods: Use molecular methods alongside traditional culture-based techniques for a comprehensive understanding of fungal diversity and its implications.
- Species-Specific Monitoring: Focus on targeting *Aspergillus fumigatus*, *Fusarium* sp., Mucorales order, *Stachybotrys atra*, and *Aspergillus* section *Flavi* due to their clinical and toxicological relevance.
- Seasonal and Geographical Variations: Explore the prevalence of these fungi across different educational environments to allow for targeted interventions and clearer guidelines.
- Regulatory Frameworks: Implement guidelines that consider both fungal species and environmental factors (e.g., humidity, ventilation, building materials). Integrate fungal monitoring into broader public health strategies.

A major limitation in this type of review is the disparate methodologies used in presenting results across various articles. Many studies offer only statistical summaries that lack comparability with others, posing significant challenges when attempting to synthesize findings or draw broader conclusions. Therefore, it is crucial to standardize the way data is presented:

- Measurement Protocols: Develop standardized measurement protocols and use unified units of measurement, such as spores per cubic meter of air, to ensure consistency.
- Meta-Analyses Frameworks: Create frameworks for meta-analyses to aggregate and standardize diverse data.
- Centralized Data Repositories: Establish centralized data repositories for sharing raw data, along with comprehensive reporting guidelines detailing study methods to facilitate comparisons.
- Interdisciplinary Collaboration: Promote interdisciplinary collaboration and secure regulatory and policy support for standardized methodologies.
- Education and Training: Provide education and training on best practices for data collection and reporting to improve the quality and comparability of research on airborne fungi and health outcomes.

Such standardization would enhance the comparability of results across studies, facilitate new research, and promote information sharing among researchers, ultimately advancing the field. Linking environmental exposure to health outcomes underscores the importance of establishing regulatory limits based on health outcomes. While there are recognized health impacts linked to airborne fungal exposure, the exact exposure-response relationships are inadequately characterized, highlighting the need for further research to address these complexities. This interconnectedness of the environment, exposure, and health outcomes emphasizes the need for comprehensive regulatory frameworks that consider these interdependencies. Ongoing research efforts aimed at elucidating the relationship between environmental fungal exposure and health outcomes will be instrumental in informing evidence-based regulatory policies to safeguard public health.

## Footnotes

^1^ https://ec.europa.eu/commission/presscorner/detail/en/ip_22_4474

## References

[1] WHO WHO fungal priority pathogens list to guide research, development and public health action. (2022). Available online at: https://www.who.int/publications-detail-redirect/9789240060241 (Accessed April 30, 2024).

[2] Seidel D Wurster S Jenks JD Sati H Gangneux J-P Egger M. Impact of climate change and natural disasters on fungal infections. Lancet Microbe. (2024) 5:9. doi: 10.1016/S2666-5247(24)00039-9, PMID: 38518791

[3] Viegas C Almeida B Aranha Caetano L Afanou A Straumfors A Veríssimo C. Algorithm to assess the presence of *Aspergillus fumigatus* resistant strains: the case of Norwegian sawmills. Int J Environ Health Res. (2022) 32:963–71. doi: 10.1080/09603123.2020.1810210, PMID: 32814444

[4] Viegas C Pena P Dias M Gomes B Cervantes R Carolino E. Microbial contamination in waste collection: unveiling this Portuguese occupational exposure scenario. J Environ Manag. (2022) 314:115086. doi: 10.1016/j.jenvman.2022.115086, PMID: 35483278

[5] Viegas C Almeida B Monteiro A Caetano LA Carolino E Gomes AQ. Bioburden in health care centers: is the compliance with Portuguese legislation enough to prevent and control infection? Build Environ. (2019) 160:106226. doi: 10.1016/j.buildenv.2019.106226

[6] Viegas C Eriksen E Gomes B Dias M Cervantes R Pena P. Comprehensive assessment of occupational exposure to microbial contamination in waste sorting facilities from Norway. Front Public Health. (2023) 11:725. doi: 10.3389/fpubh.2023.1297725, PMID: 38179569 PMC10766354

[7] ISIAQ STC34. Indoor environmental quality guidelines database. (2020). Available online at: https://ieqguidelines.org/. (Accessed May 24, 2024).

[8] Toyinbo O Hägerhed L Dimitroulopoulou S Dudzinska M Emmerich S Hemming D. Open database for international and national indoor environmental quality guidelines. Indoor Air. (2022) 32:e13028. doi: 10.1111/ina.13028, PMID: 35481936 PMC11099937

[9] IEQ Guidelines—International Society of Indoor Air Quality and Climate. (n.d.). IEQ guidelines. Available online at: https://www.isiaq.org/stc34_ieq_guidelines.php. (Accessed April 4, 2024).

[10] Du C Li B Yu W Yao R Cai J Li B. Characteristics of annual mold variations and association with childhood allergic symptoms/diseases via combining surveys and home visit measurements. Indoor Air. (2022) 32:e13113. doi: 10.1111/ina.13113, PMID: 36168229

[11] Rosen J. WHO guidelines for indoor air quality: dampness and mould. (2009). Available online at: https://www.who.int/publications/i/item/9789289041683 (Accessed April 16, 2024).

[12] WHO European Centre for Environment and Health. WHO report on school environment: policies and current status. (2015). Available online at: http://www.euro.who.int/en/media-centre/events/events/2015/04/ehp-mid-term-review/publications/the-school-environment-policies-and-current-status/_recache (Accessed April 20, 2024).

[13] Rook G a W. 99th Dahlem conference on infection, inflammation and chronic inflammatory disorders: Darwinian medicine and the ‘hygiene’ or ‘old friends’ hypothesis. Clin Exp Immunol. (2010) 160:70–9. doi: 10.1111/j.1365-2249.2010.04133.x, PMID: 20415854 PMC2841838

[14] Jartti T Korppi M. Rhinovirus-induced bronchiolitis and asthma development. Pediatr Allergy Immunol. (2011) 22:350–5. doi: 10.1111/j.1399-3038.2011.01170.x, PMID: 21535176

[15] Skevaki CL Tsialta P Trochoutsou AI Logotheti I Makrinioti H Taka S. Associations between viral and bacterial potential pathogens in the nasopharynx of children with and without respiratory symptoms. Pediatr Infect Dis J. (2015) 34:1296–301. doi: 10.1097/INF.0000000000000872, PMID: 26262821

[16] Douwes J Thorne P Pearce N Heederik D. Bioaerosol health effects and exposure assessment: progress and prospects. Ann Occup Hyg. (2003) 47:187–200. doi: 10.1093/annhyg/meg032, PMID: 12639832

[17] Eduard W. Fungal spores: a critical review of the toxicological and epidemiological evidence as a basis for occupational exposure limit setting. Crit Rev Toxicol. (2009) 39:799–864. doi: 10.3109/10408440903307333, PMID: 19863384

[18] Mendell MJ Mirer AG Cheung K Tong M Douwes J. Respiratory and allergic health effects of dampness, Mold, and dampness-related agents: a review of the epidemiologic evidence. Environ Health Perspect. (2011) 119:748–56. doi: 10.1289/ehp.1002410, PMID: 21269928 PMC3114807

[19] Harding CF Pytte CL Page KG Ryberg KJ Normand E Remigio GJ. Mold inhalation causes innate immune activation, neural, cognitive and emotional dysfunction. Brain Behav Immun. (2020) 87:218–28. doi: 10.1016/j.bbi.2019.11.006, PMID: 31751617 PMC7231651

[20] Jedrychowski W Maugeri U Perera F Stigter L Jankowski J Butscher M. Cognitive function of 6-year-old children exposed to moldcontaminated homes in early postnatal period. Prospective birth cohort study in Poland. Physiol Behav. (2011) 104:989–95. doi: 10.1016/j.physbeh.2011.06.019, PMID: 21763705 PMC3758954

[21] Deng S Zou B Lau J. The adverse associations of classrooms’ indoor air quality and thermal comfort conditions on students’ illness related absenteeism between heating and non-heating seasons—a pilot study. Int J Environ Res Public Health. (2021) 18:500. doi: 10.3390/ijerph18041500, PMID: 33562454 PMC7914660

[22] American Academy of Pediatrics. Toxic effects of indoor molds. Pediatrics. (1998) 101:712–4. 9521963

[23] Sauliene I Valiulis A Keriene I Sukiene L Dovydaityte D Prokopciuk N. Airborne pollen and fungi indoors: evidence from primary schools in Lithuania. Heliyon. (2023) 9:e12668. doi: 10.1016/j.heliyon.2022.e12668, PMID: 36685406 PMC9850001

[24] Cervantes R Dias M Gomes B Carolino E Viegas C. Development of an indexed score to identify the most suitable sampling method to assess occupational exposure to fungi. Atmosphere. (2022) 13:123. doi: 10.3390/atmos13071123

[25] Hutton B Salanti G Caldwell DM Chaimani A Schmid CH Cameron C. The PRISMA extension statement for reporting of systematic reviews incorporating network meta-analyses of health care interventions: checklist and explanations. Ann Intern Med. (2015) 162:777–84. doi: 10.7326/M14-2385, PMID: 26030634

[26] IJERPH. Associations between nature exposure and health: a review of the evidence. Int. J. Environ. Res. Public Health. (2021) 18:4790. doi: 10.3390/ijerph18094790 33946197 PMC8125471

[27] Seneviratne SI Nicholls N Easterling D Goodess C Kanae S Kossin J. Changes in climate extremes and their impacts on the natural physical environment. In: CB Field, V Barros, TF Stocker, D Qin, DJ Dokken, KL Ebi et al., editors. Managing the Risks of Extreme Events and Disasters to Advance Climate Change Adaptation. A Special Report of Working Groups I and II of the Intergovernmental Panel on Climate Change (IPCC). Cambridge, UK, and New York, NY, USA: Cambridge University Press (2012). 109–230.

[28] Ramachandran G Adgate JL Banerjee S Church TR Jones D Fredrickson A. Indoor air quality in two urban elementary schools—measurements of airborne Fungi, carpet allergens, CO2, temperature, and relative humidity. J Occup Environ Hyg. (2005) 2:553–66. doi: 10.1080/15459620500324453, PMID: 16223714

[29] Bartlett KH Kennedy SM Brauer M Van Netten C Dill B. Evaluation and a predictive model of airborne fungal concentrations in school classrooms. Ann Occup Hyg. (2004) 48:547–54. doi: 10.1093/annhyg/meh051, PMID: 15302620

[30] Pyrri I Zoma A Barmparesos N Assimakopoulos MN Assimakopoulos VD Kapsanaki-Gotsi E. Impact of a green roof system on indoor fungal aerosol in a primary school in Greece. Sci Total Environ. (2020) 719:137447. doi: 10.1016/j.scitotenv.2020.137447, PMID: 32112954

[31] Baxi SN Sheehan WJ Sordillo JE Muilenberg ML Rogers CA Gaffin JM. Association between fungal spore exposure in inner-city schools and asthma morbidity. Annals Allergy Asthma Immunol. (2019) 122:610–615.e1. doi: 10.1016/j.anai.2019.03.011, PMID: 30904580 PMC6555650

[32] Su HJ Wu PC Lin CY. Fungal exposure of children at homes and schools: a health perspective. Arch Environ Health. (2001) 56:144–9. doi: 10.1080/00039890109604066, PMID: 11339678

[33] Minahan NT Chen C-H Shen W-C Lu T-P Kallawicha K Tsai K-H. Fungal spore richness in school classrooms is related to surrounding Forest in a season-dependent manner. Microb Ecol. (2022) 84:351–62. doi: 10.1007/s00248-021-01844-2, PMID: 34498118

[34] Fonseca Gabriel M Paciência I Felgueiras F Cavaleiro Rufo J Castro Mendes F Farraia M. Environmental quality in primary schools and related health effects in children. An overview of assessments conducted in the northern Portugal. Energ Buildings. (2021) 250:111305. doi: 10.1016/j.enbuild.2021.111305

[35] Madureira J Pereira C Paciência I Teixeira JP de Oliveira Fernandes E. Identification and levels of airborne Fungi in Portuguese primary schools. J Toxic Environ Health A. (2014) 77:816–26. doi: 10.1080/15287394.2014.909302, PMID: 25072714

[36] Anedda E Traversi D. Bioaerosol in composting facilities: a survey on full-scale plants in Italy. Atmosphere. (2020) 11:398. doi: 10.3390/atmos11040398

[37] Pardo A. Chapter 6—walk-through survey In: Ribak J Rayman RB Froom P, editors. Occupational health in aviation. Amsterdam, Netherlands: Academic Press (1995). 73–115.

[38] Lindsley W. G. Green B. J. Blachere F. M. Martin S. B. Jensen P. A. Schafer M. P. (2017) Sampling and characterization of bioaerosols. In: NIOSH Manual of Analytical Methods (NMAM), 5th Edn. Washington, DC: National Institute for Occupational Safety and Health. Differentially culturable Mycobacterium tuberculosis in cough-generated aerosols of patients with pulmonary tuberculosis DCTB in cough-generated aerosols. Available at: https://www.researchgate.net/publication/392473264_Differentially_culturable_Mycobacterium_tuberculosis_in_cough-generated_aerosols_of_patients_with_pulmonary_tuberculosis_DCTB_in_cough-generated_aerosols (Accessed June 09, 2025).

[39] Herke M Moor I Winter K Hack M Hoffmann S Spallek J. Role of contextual and compositional characteristics of schools for health inequalities in childhood and adolescence: a scoping review. BMJ Open. (2022) 12:e052925. doi: 10.1136/bmjopen-2021-052925, PMID: 35105578 PMC8808395

[40] Thomas NM Calderón L Senick J Sorensen-Allacci M Plotnik D Guo M. Investigation of indoor air quality determinants in a field study using three different data streams. Build Environ. (2019) 154:281–95. doi: 10.1016/j.buildenv.2019.03.022

[41] Jacobs J Borràs-Santos A Krop E Täubel M Leppänen H Haverinen-Shaughnessy U. Dampness, bacterial and fungal components in dust in primary schools and respiratory health in schoolchildren across Europe. Occup Environ Med. (2014) 71:704–12. doi: 10.1136/oemed-2014-102246, PMID: 25035116

[42] Meklin T Husman T Vepsäläinen A Vahteristo M Koivisto J Halla-Aho J. Indoor air microbes and respiratory symptoms of children in moisture damaged and reference schools. Indoor Air. (2002) 12:175–83. doi: 10.1034/j.1600-0668.2002.00169.x, PMID: 12244747

[43] Zhang X Zhao Z Nordquist T Larsson L Sebastian A Norback D. A longitudinal study of sick building syndrome among pupils in relation to microbial components in dust in schools in China. Sci Total Environ. (2011) 409:5253–9. doi: 10.1016/j.scitotenv.2011.08.059, PMID: 21943723

[44] Foarde K Berry M. Comparison of biocontaminant levels associated with hard vs. carpet floors in nonproblem schools: results of a yearlong study. J Expo Sci Environ Epidemiol. (2004) 14:S41–8. doi: 10.1038/sj.jea.7500357, PMID: 15118744

[45] Godwin C Batterman S. Indoor air quality in Michigan schools. Indoor Air. (2007) 17:109–21. doi: 10.1111/j.1600-0668.2006.00459.x, PMID: 17391233

[46] Santilli J Rockwell W. Fungal contamination of elementary schools: a new environmental hazard. Ann Allergy Asthma Immunol. (2003) 90:203–8. doi: 10.1016/S1081-1206(10)62142-4, PMID: 12602667

[47] Mandal J Brandl H. Bioaerosols in indoor environment—a review with special reference to residential and occupational locations. Open Environ Biol Monit J. (2011) 4:83–96. doi: 10.2174/1875040001104010083

[48] Napoli C Marcotrigiano V Montagna MT. Air sampling procedures to evaluate microbial contamination: a comparison between active and passive methods in operating theatres. BMC Public Health. (2012) 12:594. doi: 10.1186/1471-2458-12-594, PMID: 22853006 PMC3444341

[49] Viegas C. Sampling methods for an accurate mycobiota occupational exposure assessment—overview of several ongoing projects In: Occupational safety and hygiene VI: CRC Press (2018)

[50] Whitby C Ferguson RMW Colbeck I Dumbrell AJ Nasir ZA Marczylo E. Chapter three—compendium of analytical methods for sampling, characterization and quantification of bioaerosols In: Bohan DA Dumbrell A, editors. Advances in ecological research, vol. 67. Amsterdam, Netherlands: Academic Press (2022). 101–229.

[51] Yasanthika W Wanasinghe D Mortimer P Monkai J Farias A. The importance of culture-based techniques in the genomic era for assessing the taxonomy and diversity of soil fungi. Mycosphere. (2022) 13:724–51. doi: 10.5943/mycosphere/13/1/8

[52] Mbareche H Veillette M Teertstra W Kegel W Bilodeau GJ Wösten HAB. Recovery of fungal cells from air samples: a tale of loss and gain. Appl Environ Microbiol. (2019) 85:e02941-18. doi: 10.1128/AEM.02941-18, PMID: 30824432 PMC6495771

[53] Peccia J Hernandez M. Incorporating polymerase chain reaction-based identification,population characterization, and quantification of microorganisms into aerosol science: a review. Atmos Environ. (2006) 40:3941–61. doi: 10.1016/j.atmosenv.2006.02.029, PMID: 32288550 PMC7108281

[54] Vashisht V Vashisht A Mondal AK Farmaha J Alptekin A Singh H. Genomics for emerging pathogen identification and monitoring: prospects and obstacles. BioMedInformatics. (2023) 3:1145–77. doi: 10.3390/biomedinformatics3040069, PMID: 40278680

[55] Viegas C Almeida B Dias M Caetano LA Carolino E Gomes AQ. Assessment of children’s potential exposure to bioburden in indoor environments. Atmos. (2020) 11:9. doi: 10.3390/atmos11090993, PMID: 40278680

[56] Haas AL Bradley BT Hanson KE. Recent developments in culture-independent fungal diagnostics. Infect Dis Clin N Am. (2025) 39:41–56. doi: 10.1016/j.idc.2024.11.004, PMID: 39701896

[57] M54. Principles and procedures for detection and culture of fungi in clinical specimens. (2025). Available online at: https://clsi.org/shop/standards/m54/ (Accessed May 12, 2025).

[58] McLain JE Cytryn E Durso LM Young S. Culture-based methods for detection of antibiotic resistance in agroecosystems: advantages, challenges, and gaps in knowledge. J Environ Qual. (2016) 45:432–40. doi: 10.2134/jeq2015.06.0317, PMID: 27065389

[59] Rastogi G Sani RK. Molecular techniques to assess microbial community structure, function, and dynamics in the environment In: Ahmad I Ahmad F Pichtel J, editors. Microbes and microbial technology: Agricultural and environmental applications. Springer, New York, NY: Springer (2011). 29–57.

[60] EUCAST. Screening for azole and echinocandin resistance. (2022). Available online at: https://www.eucast.org/eucast_news/news_singleview?tx_ttnews%5Btt_news%5D=492&cHash=f0d89d1ec647b2db54307f2be14bed98 (Accessed June 12, 2024).

[61] Garcia-Rubio R Cuenca-Estrella M Mellado E. Triazole resistance in *aspergillus* species: an emerging problem. Drugs. (2017) 77:599–613. doi: 10.1007/s40265-017-0714-4, PMID: 28236169

[62] Raja HA Miller AN Pearce CJ Oberlies NH. Fungal identification using molecular tools: a primer for the natural products research community. J Nat Prod. (2017) 80:756–70. doi: 10.1021/acs.jnatprod.6b01085, PMID: 28199101 PMC5368684

[63] Houbraken J Kocsubé S Visagie CM Yilmaz N Wang X-C Meijer M. Classification of aspergillus, Penicillium, Talaromyces and related genera (Eurotiales): an overview of families, genera, subgenera, sections, series and species. Stud Mycol. (2020) 95:5–169. doi: 10.1016/j.simyco.2020.05.002, PMID: 32855739 PMC7426331

[64] Mbareche H Veillette M Bilodeau GJ. In silico study suggesting the bias of primers choice in the molecular identification of fungal aerosols. J Fungi. (2021) 7:99. doi: 10.3390/jof7020099, PMID: 33573216 PMC7911573

[65] Lamoth F. *Aspergillus fumigatus*-related species in clinical practice. Front Microbiol. (2016) 7:683. doi: 10.3389/fmicb.2016.00683, PMID: 27242710 PMC4868848

[66] Park H-S Jun S-C Han K-H Hong S-B Yu J-H. Diversity, application, and synthetic biology of industrially important aspergillus Fungi. Adv Appl Microbiol. (2017) 100:161–202. doi: 10.1016/bs.aambs.2017.03.001, PMID: 28732553

[67] Nargesi S Jafarzadeh J Najafzadeh MJ Nouripour-Sisakht S Haghani I Abastabar M. Molecular identification and antifungal susceptibility of clinically relevant and cryptic species of aspergillus sections Flavi and Nigri. J Med Microbiol. (2022) 71:480. doi: 10.1099/jmm.0.001480, PMID: 35451946

[68] Sabino R Gonçalves P Martins Melo A Simões D Oliveira M Francisco M. Trends on Aspergillus epidemiology-perspectives from a national reference laboratory surveillance program. Journal of Fungi (Basel, Switzerland). (2021) 7:28. doi: 10.3390/jof7010028 PMC7825284 33418997

[69] Assunção R Martins C Viegas S Viegas C Jakobsen LS Pires S. Climate change and the health impact of aflatoxins exposure in Portugal—an overview. Food Addit Contam Part A Chem Anal Control Expo Risk Assess. (2018) 35:1610–21. doi: 10.1080/19440049.2018.1447691, PMID: 29494310

[70] Benkerroum N. Aflatoxins: producing-molds, structure, health issues and incidence in southeast Asian and sub-Saharan African countries. Int J Environ Res Public Health. (2020) 17:1215. doi: 10.3390/ijerph17041215, PMID: 32070028 PMC7068566

[71] Leggieri MC Toscano P Battilani P. Predicted aflatoxin B1 increase in Europe due to climate change: actions and reactions at global level. Toxins. (2021) 13:292. doi: 10.3390/toxins13040292, PMID: 33924246 PMC8074758

[72] European Environment Agency. Europe’s changing climate hazards—an index-based interactive EEA report. (2022). Available online at: https://www.eea.europa.eu/publications/europeschanging-climate-hazards-1/climate-hazards-indices (Accessed April 4, 2025).

[73] Page MJ McKenzie JE Bossuyt PM Boutron I Hoffmann TC Mulrow CD. The PRISMA 2020 statement: an updated guideline for reporting systematic reviews. BMJ. (2021) 372:n71. doi: 10.1136/bmj.n71 33782057 PMC8005924

[74] Baxi SN Muilenberg ML Rogers CA Sheehan WJ Gaffin J Permaul P. Exposures to molds in school classrooms of children with asthma. Pediatr Allergy Immunol. (2013) 24:697–703. doi: 10.1111/pai.12127, PMID: 24112429 PMC3782748

[75] Chen B-Y Jasmine Chao H Wu C-F Kim H Honda Y Guo YL. High ambient Cladosporium spores were associated with reduced lung function in schoolchildren in a longitudinal study. Sci Total Environ. (2014) 481:370–6. doi: 10.1016/j.scitotenv.2014.01.078, PMID: 24607630

[76] Cavaleiro Rufo J Madureira J Paciência I Aguiar L Pereira C Silva D. Indoor fungal diversity in primary schools may differently influence allergic sensitization and asthma in children. Pediatr Allergy Immunol. (2017) 28:332–9. doi: 10.1111/pai.12704, PMID: 28208225

[77] Mentese S Arisoy M Rad AY Güllü G. Bacteria and fungi levels in various indoor and outdoor environments in Ankara, Turkey. CLEAN Soil Air Water. (2009) 37:487–93. doi: 10.1002/clen.200800220

[78] Bartlett KH Kennedy SM Brauer M van Netten C Dill B. Evaluation and determinants of airborne bacterial concentrations in school classrooms. J Occup Environ Hyg. (2004) 1:639–47. doi: 10.1080/15459620490497744, PMID: 15631055

[79] Yamamoto N Hospodsky D Dannemiller KC Nazaroff WW Peccia J. Indoor emissions as a primary source of airborne allergenic fungal particles in classrooms. Environ Sci Technol. (2015) 49:5098–106. doi: 10.1021/es506165z, PMID: 25794178

[80] Zhang G Neumeister-Kemp H Garrett M Kemp P Stick S Franklin P. Exposure to airborne mould in school environments and nasal patency in children. Indoor Built Environ. (2013) 22:608–17. doi: 10.1177/1420326X12447534

[81] Meklin T Hyvärinen A Toivola M Reponen T Koponen V Husman T. Effect of building frame and moisture damage on microbiological indoor air quality in school buildings. AIHA J. (2003) 64:108–16. doi: 10.1080/15428110308984800, PMID: 12570403

[82] Putus T Tuomainen A Rautiala S. Chemical and microbial exposures in a school building: adverse health effects in children. Arch Environ Health. (2004) 59:194–201. doi: 10.3200/AEOH.59.4.194-201, PMID: 16189992

[83] Awad AH Saeed Y Hassan Y Fawzy Y Osman M. Air microbial quality in certain public buildings, Egypt: a comparative study. Atmospheric Pollut Res. (2018) 9:617–26. doi: 10.1016/j.apr.2017.12.014

[84] Straus DC Cooley JD Wong WC Jumper CA. Studies on the role of Fungi in sick building syndrome. Arch Environ Health. (2003) 58:475–8. doi: 10.3200/AEOH.58.8.475-478, PMID: 15259426

[85] Holst GJ Høst A Doekes G Meyer HW Madsen AM Plesner KB. Allergy and respiratory health effects of dampness and dampness-related agents in schools and homes: a cross-sectional study in Danish pupils. Indoor Air. (2016) 26:880–91. doi: 10.1111/ina.12275, PMID: 26643593

[86] Würtz H Sigsgaard T Valbjørn O Doekes G Meyer HW. The dustfall collector—a simple passive tool for long-term collection of airborne dust: a project under the Danish Mould in buildings program (DAMIB). Indoor Air. (2005) 15:33–40. doi: 10.1111/j.1600-0668.2005.00342.x, PMID: 15910527

[87] Kim JL Elfman L Mi Y Wieslander G Smedje G Norbäck D. Indoor molds, bacteria, microbial volatile organic compounds and plasticizers in schools—associations with asthma and respiratory symptoms in pupils. Indoor Air. (2007) 17:153–63. doi: 10.1111/j.1600-0668.2006.00466.x, PMID: 17391238

[88] Simoni M Cai G-H Norback D Annesi-Maesano I Lavaud F Sigsgaard T. Total viable molds and fungal DNA in classrooms and association with respiratory health and pulmonary function of European schoolchildren. Pediatr Allergy Immunol. (2011) 22:843–52. doi: 10.1111/j.1399-3038.2011.01208.x, PMID: 22122789

[89] Shin S-K Kim J Ha S Oh H-S Chun J Sohn J. Metagenomic insights into the bioaerosols in the indoor and outdoor environments of childcare facilities. PLoS One. (2015) 10:e0126960. doi: 10.1371/journal.pone.0126960, PMID: 26020512 PMC4447338

[90] Nieto-Caballero M Gomez OM Shaughnessy R Hernandez M. Aerosol fluorescence, airborne hexosaminidase, and quantitative genomics distinguish reductions in airborne fungal loads following major school renovations. Indoor Air. (2022) 32:e12975. doi: 10.1111/ina.12975, PMID: 34897813

[91] Celtik C Okten S Okutan O Aydogdu H Bostancioglu M Ekuklu G. Investigation of indoor molds and allergic diseases in public primary schools in Edirne city of Turkey. Asian Pac J Allergy Immunol. (2011) 29:42–9. 21560487

[92] Park J-H Lemons AR Croston TL Park Y Roseman J Green BJ. Mycobiota and the contribution of yeasts in floor dust of 50 elementary schools characterized with sequencing internal transcribed spacer region of ribosomal DNA. Environ Sci Technol. (2022) 56:11493–503. doi: 10.1021/acs.est.2c01703, PMID: 35901271 PMC10183301

[93] Chatzidiakou L Mumovic D Summerfield AJ Hong SM Altamirano-Medina H. A victorian school and a low carbon designed school: comparison of indoor air quality, energy performance, and student health. Indoor Built Environ. (2014) 23:417–32. doi: 10.1177/1420326X14532388

[94] Hanson B Zhou Y Bautista EJ Urch B Speck M Silverman F. Characterization of the bacterial and fungal microbiome in indoor dust and outdoor air samples: a pilot study. Environ Sci: Processes Impacts. (2016) 18:713–24. doi: 10.1039/C5EM00639B, PMID: 27213188 PMC5015483

[95] Lee BG Yang JI Kim E Geum SW Park J-H Yeo M-K. Investigation of bacterial and fungal communities in indoor and outdoor air of elementary school classrooms by 16S rRNA gene and ITS region sequencing. Indoor Air. (2021) 31:1553–62. doi: 10.1111/ina.12825, PMID: 33780050 PMC10230515

[96] Huttunen K Wlodarczyk AJ Tirkkonen J Mikkonen S Täubel M Krop E. Oxidative capacity and hemolytic activity of settled dust from moisture-damaged schools. Indoor Air. (2019) 29:299–307. doi: 10.1111/ina.12527, PMID: 30575131

[97] Vornanen-Winqvist C Järvi K Andersson MA Duchaine C Létourneau V Kedves O. Exposure to indoor air contaminants in school buildings with and without reported indoor air quality problems. Environ Int. (2020) 141:105781. doi: 10.1016/j.envint.2020.105781, PMID: 32417615

[98] Al-Qurashi A. Profiles of airborne fungi in schools of Saudi Arabia in relation to the allergy problems and respiratory diseases. J Food Agric Environ. (2007) 5

[99] Andualem Z Gizaw Z Dagne H. Indoor culturable fungal load and associated factors among public primary school classrooms in Gondar City, Northwest Ethiopia, 2018: a crosssectional study. Ethiop J Health Sci. (2019) 29:13. doi: 10.4314/ejhs.v29i5.13 PMC6813266 31666784

[100] Aydogdu H Asan A Otkun MT Ture M. Monitoring of fungi and bacteria in the indoor air of primary schools in Edirne City, Turkey. Indoor Built Environ. (2005) 14:411–25. doi: 10.1177/1420326X05057539

[101] Hyvonen SM Lohi JJ Rasanen LA Heinonen T Mannerstrom M Vaali K. Association of toxic indoor air with multi-organ symptoms in pupils attending a moisture damaged school in Finland. Am J Clin Exp Immunol. (2020) 9:101–13. 33489478 PMC7811924

[102] Ejdys E Biedunkiewicz A. Fungi of the genus *Penicillium* in school buildings. Polish J Environ Stud. (2011) 20:333–8.

[103] Savilahti R Uitti J Roto P Laippala P Husman T. Increased prevalence of atopy among children exposed to mold in a school building. Allergy. (2001) 56:175–9. doi: 10.1034/j.1398-9995.2001.056002175.x, PMID: 11167380

[104] PEO Model. Available online at: https://peomodel.com/#:~:text=The%20Person%2DEnvironment%2DOccupation%20(,and%20Lori%20Letts%20in%201996 (1996). (Accessed June 5, 2024).

[105] Vornanen-Winqvist C Salonen H Järvi K Andersson MA Mikkola R Marik T. Effects of ventilation improvement on measured and perceived indoor air quality in a school building with a hybrid ventilation system. Int J Environ Res Public Health. (2018) 15:414. doi: 10.3390/ijerph15071414, PMID: 29976864 PMC6068750

[106] Law M Cooper B Strong S Stewart D Rigby P Letts L. The person-environment-occupation model: a transactive approach to occupational performance. Canadian Journal of Occupational Therapy. (1996) 63:9–23. doi: 10.1177/000841749606300103 10462885

[107] Moher D Liberati A Tetzlaff J Altman DG PRISMA Group (2009). Preferred reporting items for systematic reviews and meta-analyses: the PRISMA statement. PLoS medicine, 6:e1000097. doi: 10.1371/journal.pmed.1000097 19621072 PMC2707599

